# Urine Exosomes for Non-Invasive Assessment of Gene Expression and Mutations of Prostate Cancer

**DOI:** 10.1371/journal.pone.0154507

**Published:** 2016-05-04

**Authors:** Piruz Motamedinia, Anna N. Scott, Kendall L. Bate, Neda Sadeghi, Guillermo Salazar, Edan Shapiro, Jennifer Ahn, Michael Lipsky, James Lin, Greg W. Hruby, Ketan K. Badani, Daniel P. Petrylak, Mitchell C. Benson, Michael J. Donovan, Wayne D. Comper, James M. McKiernan, Leileata M. Russo

**Affiliations:** 1 Department of Urology, Columbia University Medical Center, Department of Urology, New York, New York, United States of America; 2 Exosome Diagnostics, Inc. New York, New York, United States of America; 3 Department of Pathology, Icahn School of Medicine at Mt. Sinai, New York, New York, United States of America; University of Kentucky College of Medicine, UNITED STATES

## Abstract

**Purpose:**

The analysis of exosome/microvesicle (extracellular vesicles (EVs)) and the RNA packaged within them (exoRNA) has the potential to provide a non-invasive platform to detect and monitor disease related gene expression potentially *in lieu* of more invasive procedures such as biopsy. However, few studies have tested the diagnostic potential of EV analysis in humans.

**Experimental Design:**

The ability of EV analysis to accurately reflect prostate tissue mRNA expression was examined by comparing urinary EV TMPRSS2:ERG exoRNA from pre-radical prostatectomy (RP) patients versus corresponding RP tissue in 21 patients. To examine the differential expression of TMPRSS2:ERG across patient groups a random urine sample was taken without prostate massage from a cohort of 207 men including prostate biopsy negative (Bx Neg, n = 39), prostate biopsy positive (Bx Pos, n = 47), post-radical prostatectomy (post-RP, n = 37), un-biopsied healthy age-matched men (No Bx, n = 44), and young male controls (Cont, n = 40). The use of EVs was also examined as a potential platform to non-invasively differentiate Bx Pos versus Bx Neg patients via the detection of known prostate cancer genes TMPRSS2:ERG, BIRC5, ERG, PCA3 and TMPRSS2.

**Results:**

In this technical pilot study urinary EVs had a sensitivity: 81% (13/16), specificity: 80% (4/5) and an overall accuracy: 81% (17/21) for non-invasive detection of TMPRSS2:ERG versus RP tissue. The rate of TMPRSS2:ERG exoRNA detection was found to increase with age and the expression level correlated with Bx Pos status. Receiver operator characteristic analyses demonstrated that various cancer-related genes could differentiate Bx Pos from Bx Neg patients using exoRNA isolated from urinary EVs: BIRC5 (AUC 0.674 (CI:0.560–0.788), ERG (AUC 0.785 (CI:0.680–0.890), PCA3 (AUC 0.681 (CI:0.567–0.795), TMPRSS2:ERG (AUC 0.744 (CI:0.600–0.888), and TMPRSS2 (AUC 0.637 (CI:0.519–0.754).

**Conclusion:**

This pilot study suggests that urinary EVs have the potential to be used as a platform to non-invasively differentiate patients with prostate cancer with very good accuracy. Larger studies are needed to confirm the potential for clinical utility.

## Introduction

Exosomes and microvesicles are lipid bilayer vesicles (usually 30–200 nm in diameter). During formation, exosomes and microvesicles (collectively referred to herein as extracellular vesicles (EVs)) encapsulate a portion of the parent cell cytoplasm including membrane proteins, cytosolic proteins [1] and nucleic acids (chiefly RNA referred to herein as exoRNA) [2,3]. The EVs are then released from cells and may be harvested from biofluids including blood and urine. To date there have been few substantial studies to understand the potential diagnostic use of EVs.

Prostate cancer represents the most common cancer in men and the second most common cause of cancer-related death in the United States [4]. Serum prostate specific antigen (PSA) is used as a biomarker for prostate cancer although this strategy has been criticized due to its low sensitivity and specificity [5]. Schröder *et al* [6] found that 1055 men need to be screened and 37 men would need to be treated in order to save one life. This over-diagnosis and overtreatment results in decreased quality of life and increased healthcare costs, highlighting the urgent need for more sensitive and specific diagnostic strategies [7,8]. In 2009, it was reported that prostate-related genes could be successfully detected in urinary EVs [9]. We have recently advanced methods to i) rapidly isolate intact EVs removing reliance on ultracentrifugation and enabling adaptation to the clinical laboratory setting and ii) obtain high integrity exoRNA important for reliable transcriptional analysis [10,11].

In 2005, Tomlins *et al*. [12] reported the identification of the prostate specific TMPRSS2:ERG fusion mutation between the androgen driven gene transmembrane protease serine 2 (TMPRSS2) and the oncogene Ets Related Gene (ERG). The most common fusion event (TMPRSS2 exon 1 with ERG exon 4) has been reported as having an incidence between 20%-80% in prostate cancer [13,14]. Fusion status has been examined via various techniques including fluorescence *in situ* hybridization (FISH) and RT-PCR [15]. Another gene commonly examined in prostate cancer is PCA3, originally named Differential Display code 3 (DD3), PCA3 is a non-coding gene highly expressed in prostate cancer [16]. Other genes that have been implicated in prostate cancer include baculoviral IAP repeat containing 5 (BIRC5), also known as survivin [17] and ERG [18]. Kallikrein 3 (KLK3), also known to encode PSA [19], is a prostate related gene whose mRNA levels have previously been utilized to standardize gene expression in urinary sediments [20].

Previous studies examining prostate marker gene expression in urine have required prostate massage to obtain enough cells for RNA analysis. The specifics of the massage vary widely among the studies, from the application of mild digital pressure to the lateral lobes to firm pressure over the entire gland [20–26]. Such manipulations may result in patient discomfort and inter-provider variability. Additionally, the requisite prostate massage prior to urine collection mandates a direct interaction with a physician via an office visit prior to each specimen collection, resulting in increased cost. The current pilot study examines the potential diagnostic utility of EVs in a random urine sample taken without prior prostate massage. We use the detection of the prostate specific gene fusion event (TMPRSS2:ERG) in urinary EVs prior to radical prostatectomy (RP) compared with matched tissue from the surgical specimen to examine the ability of urinary EVs to reflect tissue expression. We further examine the use of EVs as a platform to non-invasively differentiate men with biopsy proven prostate cancer (Bx Pos) from those with negative prostate biopsies (Bx Neg) using previously described genes implicated in prostate cancer.

## Materials and Methods

### Sample procurement

The Columbia University Institutional Review Board approved this study. Written consent was obtained for the samples collected. Random urine samples collected without prostate massage, were obtained from 207 men grouped into: biopsy negative (Bx Neg, n = 39), biopsy positive (BX Pos, n = 47), post-radical prostatectomy with no evidence of remaining disease (post-RP, n = 37), age matched males (no history of prostate cancer) (No Bx, n = 44) and healthy controls (men <35 years old) (Cont, n = 40). Urine samples were stored at 4°C until use. Of note, the Bx Neg group included patients with high-grade prostatic intraepithelial neoplasia (HGPIN) and/or atypical small acinar proliferation (ASAP) on biopsy. All urine samples were provided for analysis consecutively and in a blinded fashion. Radical prostatectomy tissue was obtained from 21 patients for whom matching urine samples collected prior to RP were available (n = 21). The formalin fixed paraffin embedded RP tissue was cut at 5 μm and mounted onto glass slides, as per the Pathology Department protocol. The tissue was then frozen at -80°C until use.

### Urine sample processing

Urine samples were processed as previously reported [10] using 20 mL urine samples. A 0.8 μm filter (Nalgene, NY) was used to remove whole cells and cellular debris. Microvesicles/exosomes were isolated using a 100k MWCO filtration concentrator (Millipore, MA) as previously described (10). Following the addition of the urine sample, the filtration concentrator was centrifuged at 4,500 × *g* for 5 minutes and the filtrate discarded and the retentate kept. The retentate then underwent a 10 ml PBS wash and two 15ml PBS washes followed by centrifugation at 4,500 × *g* for 5 minutes after the addition of each PBS wash. 4 μl of RNasin (Promega, WI) was added to the isolated EVs prior to RNA extraction to further remove any RNase activity. exoRNA was isolated from the washed EV fraction using the RNeasy PLUS kit (Qiagen, MD) according to the Manufacturer’s instructions, which utilizes a DNA spin column for the removal of genomic DNA (gDNA) from the sample.

### Tissue processing

Patients with pre-radical prostatectomy urines were chosen at random to examine the concordance between TMPRSS2:ERG detection in the urine and detection in tissue. RP tissue was chosen so that a thorough analysis of multiple tumor foci and normal tissue regions could be analyzed. Formalin fixed paraffin embedded (FFPE) tissue from tumor foci and normal prostate areas was sectioned at 5 μm and placed on glass slides. A total of 4–6 slides from each tumor focus or normal area were compiled and nucleic acids were isolated using the QIAamp DNA FFPE tissue kit (Qiagen, CA) according to the manufacturer’s instructions. In brief, tissue sections were scraped into an Eppendorf tube in an RNase free environment. To dewax tissue sections, 1.5 mL Histology Grade Xylene (VWR International, PA) was added to the sections, tubes were briefly vortexed and spun at 20,000 × g for two minutes at room temperature. Supernatant was removed by pipette. Samples were similarly vortexed and spun following the addition of 1.5 mL, then 1 mL of 100% Ethanol (Electron Microscopy Sciences, PA). Pellets were fully dried by incubating uncapped tubes at 37°C for 20 minutes. 180 μL Buffer ALT was added directly to the pellets, which were fully homogenized using a tissue-rupture device. 20 μL Proteinase K was added and mixed with the samples, which were then incubated for 1 hour at 56°C, then 1 hour at 90°C. Tubes were briefly spun and samples were transferred to QIAamp MinElute columns in 2 mL collection tubes. Following a 1 minute 6000 × g spin, the flow through were discarded and columns were transferred to fresh tubes. Columns were spun twice at 6000 × g for 1 minute following the addition of 500 μL AW1 Buffer and 500 μL AW2 Buffer. To remove all ethanol, columns were spun at >20,000 × g for 3 minutes. Nucleic acids were eluted from columns in 35 μL of Buffer ATE, the concentration of which was measured by Nanodrop Spectrophotometer according to the manufacturer’s instructions where buffer ATE (Qiagen, CA) was used as a blank.

### PCR analysis

RNA from both tissue and urine samples underwent reverse transcriptase reaction (RT) using the VILO RT kit (Invitrogen, CA) according to manufacturer’s instructions. Sample pre-amplification was carried out using the TaqMan^®^ PreAmp Master Mix Kit (Life Technologies, NY) according to the manufacturer’s instructions. Pre-amplified cDNA was diluted 1:20 in TE buffer. PCR was performed with 5 μL of diluted, pre-amplified cDNA for the detection of TMPRSS2:ERG (Life Technologies, NY)(Hs03063375_ft, 106bp), KLK3 (PSA) (Hs03063374_m1, 64bp), ERG (Hs01554635_m1, 104bp), BIRC5 (Hs00153353_m1, 93bp) and PCA3 (Hs01371938_m1, 80bp), TMPRSS2 (Hs00237175_m1) on a StepOne Plus machine (Applied Biosystems, CA). For exoRNA gene expression, genes of interest were normalized to exoRNA KLK3 (C_t gene of interest_ − C_t KLK3_). For tissue gene expression genes of interest were normalized to GAPDH (C_t gene of interest_ − C_t GAPDH_).

### Data Analysis

The DataAssist program Version 2.0 (Applied Biosystems, CA) was used for data analysis to determine relative quantitation (RQ) of gene expression and significance of expression (*P*-value). Comparison of clinical and patient characteristics including box plots and ROC analysis was performed using STATA IC 12.1 (StataCorp, College Station, TX).

### Statistical analysis

A Student’s t-test, one-way ANOVA and ROC analyses were used to test for statistical significance. *P*<0.05 was considered significant.

## Results

**Fig 1:**
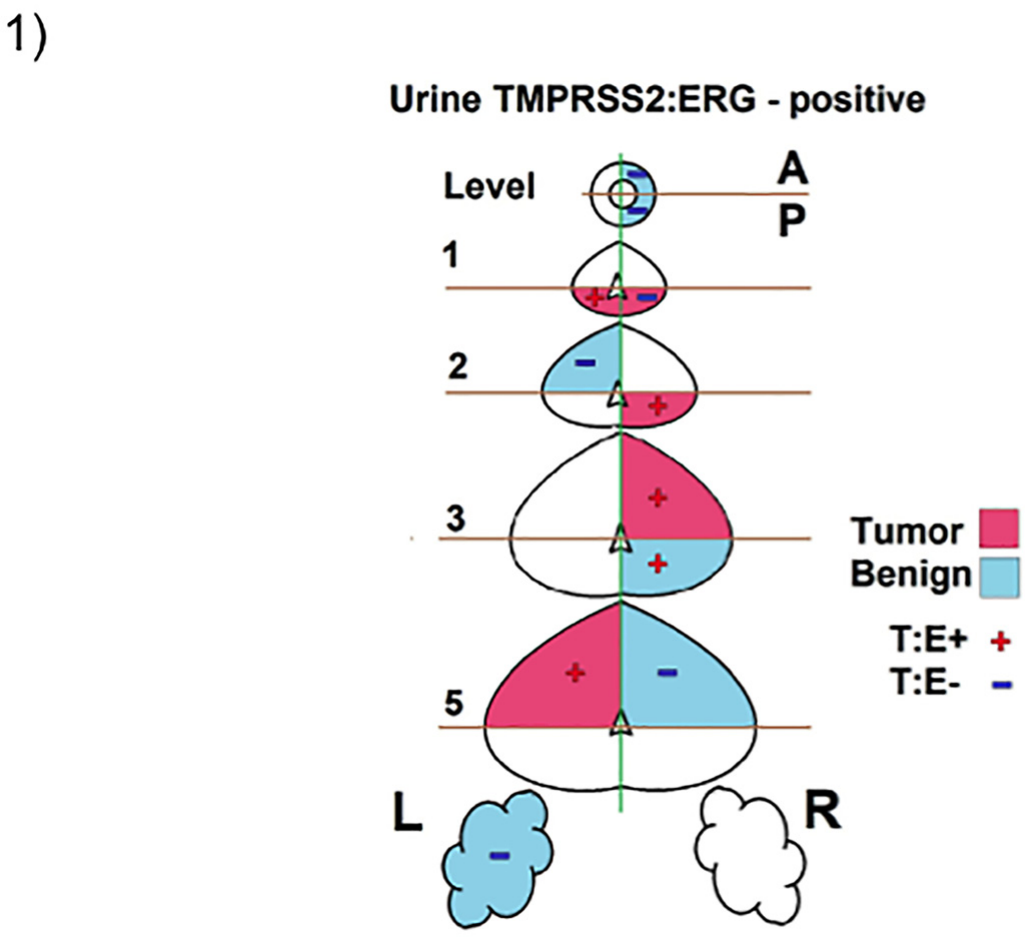
Tissue Map Detailing Pathology Determined Benign and Tumor Areas of a Prostatectomy and the Detection of TMPRSS2:ERG mRNA. Representative cartoon showing the selection of benign (blue) and cancer (pink) regions from a radical prostatectomy. The tumor and benign regions were examined for TMPRSS2:ERG mRNA expression (Red ‘+’ indicates positive TMPRSS2:ERG expression, Blue ‘-’ indicates no TMPRSS2:ERG expression). T:E—TMPRSS2:ERG, A—anterior, P—posterior, L—left, R—right.

To determine the accuracy with which exoRNA isolated from urinary EVs reflects prostate tissue mRNA, we analyzed TMPRSS2:ERG expression in RP tissue of 21 patients and compared it to matching urine samples taken prior to RP. Radical prostatectomy tissue was used (as opposed to biopsy tissue) so that a more accurate analysis could be made of TMPRSS2:ERG expression throughout the prostate. A representative illustration of the prostate areas extracted for analysis (a ‘tissue map’) of the prostatectomy tissue, the pathological status of each area (tumor or benign) and the TMPRSS2:ERG status of each area extracted is shown in Fig 1 demonstrating the extensive amount of tissue analyzed (see S1 Fig for the ‘tissue maps’ of all 21 patients). The isolation of mRNA from FFPE tissue was confirmed via the analysis of qPCR amplicons with and without prior RT reaction (see S2 Fig). In some cases, TMPRSS2:ERG expression was detected in ‘benign’ tissue regions. This may be due to the presence of micro foci of tumor in subsequent sections of the tissue extracted for RNA analysis below that originally analyzed by the pathologist (See S3 Fig) and may explain why some ‘benign’ regions unexpectedly showed positive TMPRSS2:ERG expression. A patient was considered TMPRSS2:ERG positive if TMPRSS2:ERG was detected within any of the sections of the prostatectomy tissue using RT-qPCR. This analysis was compared to TMPRSS2:ERG expression in urinary EVs using the same RT-qPCR reaction and reported in Table 1. The analysis of the detection of TMPRSS2:ERG in tissue versus detection in urinary EVs demonstrated a very good correlation with a sensitivity of 81% (13/16), specificity of 80% (4/5), PPV 93%, NPV 57% and an overall accuracy of 81% (17/21) (see Table 2). To determine why expression in urinary EVs did not always reflect tissue expression, we compared the tissue expression levels (relative quantitation (RQ)) of TMPRSS2:ERG for those patients with detectable urinary EV TMPRSS2:ERG expression versus those patients with undetectable urinary EV TMPRSS2:ERG expression. Although limited in sample size with only 3 TMPRSS2:ERG positive tissue samples being TMPRSS2:ERG negative in urinary exoRNA, versus 13 that had exact correlation with positive expression in both tissue and urinary EVs, data revealed that TMPRSS2:ERG expression in tumor tissue was ~98 fold higher (*P* = 0.009) in patients with detectable urinary exoRNA TMPRSS2:ERG expression. In another instance, TMPRSS2:ERG was detected in the urinary exoRNA but was undetectable in the limited tissue available for analysis (see Table 1, patient 10).

**Table 1:** The Detection of TMPRSS2:ERG in Urinary EVs versus Corresponding Prostatectomy Tissue from the same patients.

Patient	Urine T:E	Tissue T:E	Concordance
1	+	+	yes
2	-	-	yes
3	+	+	yes
4	-	+	no
5	+	+	yes
6	-	-	yes
7	+	+	yes
8	+	+	yes
9	-	-	yes
10	+	-	no
11	+	+	yes
12	+	+	yes
13	+	+	yes
14	+	+	yes
15	-	-	yes
16	+	+	yes
17	-	+	no
18	-	+	no
19	+	+	yes
20	+	+	yes
21	+	+	yes

T:E, TMPRSS2:ERG; +, T:E present; -, T:E absent; ‘yes’, both urine and tissue expression present; ‘no’, expression in urine or tissue.

**Table 2:** Sensitivity, specificity, PPV, NPV and accuracy for tissue versus urinary EV TMPRSS2:ERG detection.

	Biopsy T:E +	Biopsy T:E -	
**Urinary EV T:E +**	13	1	PPV 93%
**Urinary EV T:E -**	3	4	NPV 57%
	Sensitivity 81%	Specificity 80%	Accuracy 81%

T:E, TMPRSS2:ERG; +, T:E present; -, T:E absent; PPV, positive predictive value; NPV, negative predictive value.

To further examine the use of EVs to detect TMPRSS2:ERG expression, random urine samples were collected from 207 men stratified into 5 groups: Transrectal ultrasound (TRUS) prostate biopsy negative (Bx Neg, n = 39), TRUS biopsy positive (Bx Pos, n = 47), post-radical prostatectomy (post-RP, n = 37), age matched men who did not undergo biopsy (No Bx, n = 44) and controls (males under the age of 35 years; Cont, n = 40). Patient characteristics are presented in Table 3. There was no difference in mean age except for control males (*P*<0.05). Serum PSA was only significantly reduced in the post-RP group (*P*<0.001) consistent with prostate removal.

**Table 3:** Patient Characteristics.

	Bx Neg	Bx Pos	Post-RP	No Bx	Control
***n***	39	47	37	44	40
**Age**					
Mean ± SD	68 ± 6.4	69 ±8.7	65 ± 8.3	72 ± 8.8	27.9 ± 3.2*
**PSA (ng/mL)**					
Mean ± SD	5.31 ± 3.89	6.65 ± 4.93	0.00 ± 0.00^#^	2.90 ± 1.80	ND
**Ethnicity**					
White	30 (77%)	19 (45%)	29 (76%)	31 (74%)	ND
Black	0 (0%)	1 (2%)	4 (11%)	0 (0%)	ND
Hispanic	2 (5%)	7 (17%)	4 (11%)	0 (0%)	ND
Other	7 (18%)	15 (36%)	1 (2%)	11 (26%)	ND
**Clinical Stage**					
T1	-	42(86%)	29 (76%)	-	-
T2	-	7 (14%)	9 (24%)	-	-
T3	-	0 (0%)	0 (0%)	-	-
**Biopsy Grade**					
Gl<7	-	24 (52%)	10 (29%)	-	-
Gl = 7	-	19 (41%)	19 (54%)	-	-
Gl>7	-	3 (7%)	6 (17%)	-	-
**Path Stage**					
pT2	-	11 (79%)	24 (71%)	-	-
pT3	-	3 (21%)	10 (29%)	-	-
pT4	-	0 (0%)	0 (0%)	-	-
**Path Grade**					
Gl<7	-	1 (8%)	6 (17%)	-	-
Gl = 7	-	10 (77%)	26 (72%)	-	-
Gl>7	-	2 (15%)	4 (11%)	-	-

All groups were age matched except for males <35 years old (* *P*<0.05). Serum PSA was not significantly different between biopsy positive and biopsy negative patients but was significantly less in the no biopsy group (# *P*<0.05).

**Fig 2:**
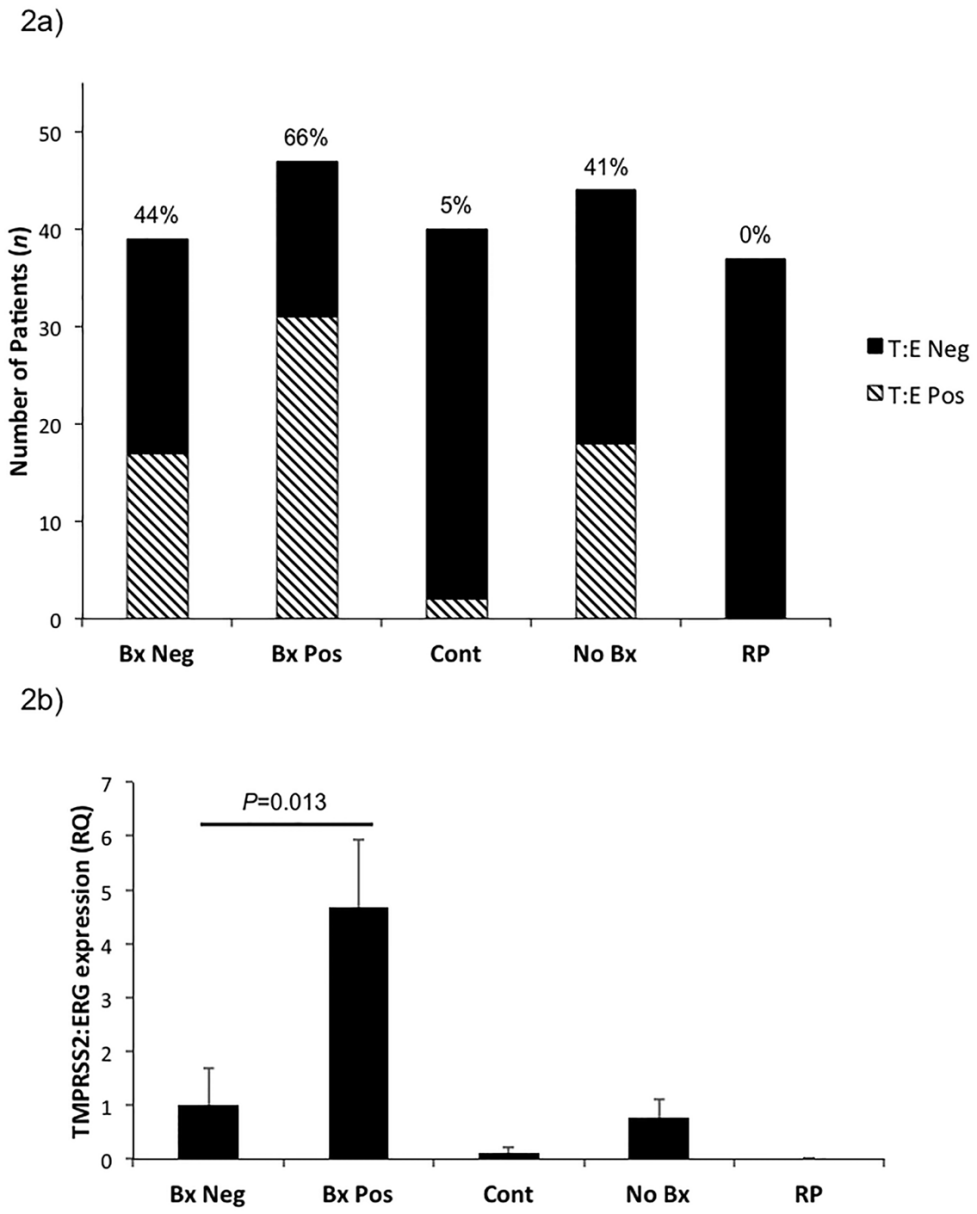
Non-invasive Analysis of the Incidence of TMPRSS2:ERG Fusion using Urinary EV exoRNA. a) Rate of TMPRSS2:ERG detection in urinary EVs. Lined bars—TMPRSS2:ERG positive, solid bars—no TMPRSS2:ERG expression. Percentage refers to TMPRSS2:ERG positive patients. b) Relative quantitation (RQ) of TMPRSS2:ERG expression versus Bx Neg patients. TMPRSS2:ERG expression was standardized to KLK3 consistent with other prostate biomarker studies [20]. Bx Neg—biopsy negative (n = 39), Bx Pos—biopsy positive (n = 47), Cont—control males <35 years old (n = 40), No Bx—no biopsy (age matched control) (n = 44), RP—radical prostatectomy (n = 37). Data presented as RQ ± SEM.

The incidence of TMPRSS2:ERG expression (Fig 2a) was highest in the Bx Pos group with 66% expressing TMPRSS2:ERG consistent with previous findings [12–14]. Interestingly, 44% of Bx Neg patients were found to express TMPRSS2:ERG. Post-RP patients had no TMPRSS2:ERG expression consistent with the prostate-specific expression of the gene, which is lost upon prostate removal [12]. Only 5% of control males (<35 yrs old) expressed TMPRSS2:ERG in contrast to 41% in ‘age-matched’ No Bx controls (males of similar age to the Bx Pos and Bx Neg groups but who did not have an indication for prostate biopsy). We further examined the level of TMPRSS2:ERG expression by RQ of TMPRSS2:ERG across groups (Fig 2b). TMPRSS2:ERG expression was highest in the Bx Pos group (*P* = 0.013) consistent with the confirmed presence of cancer (see S4 Fig for box plot distribution of delta C_t_). We also followed urinary TMPRSS2:ERG detection in patients pre and post-RP, when available. Although limited in sample size, all 3 patients who were exoRNA TMPRSS2:ERG positive prior to RP were found to have no exoRNA TMPRSS2:ERG expression post-RP, suggesting that the TMPRSS2:ERG detected in these urinary EVs was indeed prostate-specific.

**Fig 3:**
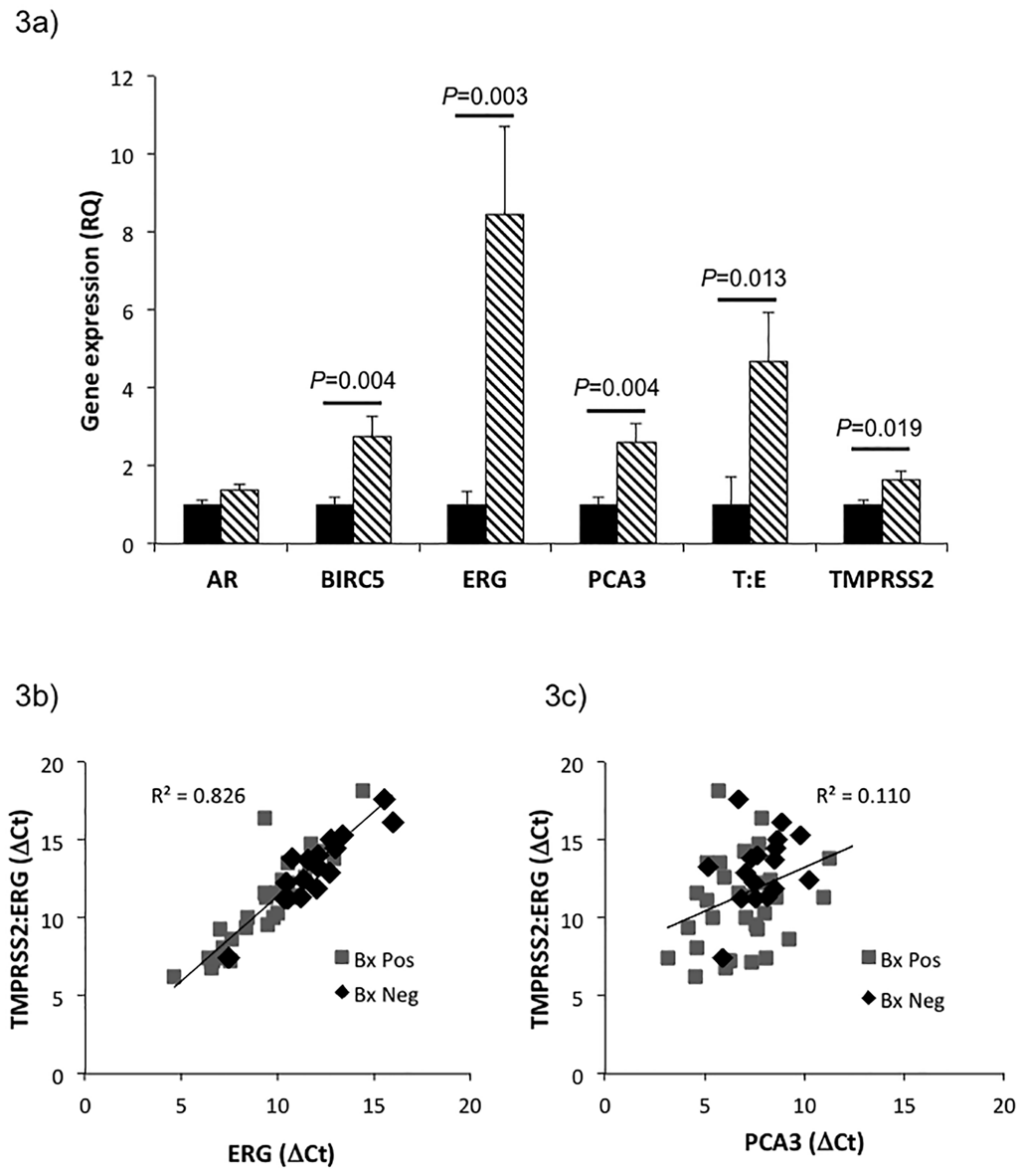
Urinary EV exoRNA Analysis can be used to Differentiate Prostate Cancer Patients. a) Relative quantitation (RQ) of gene expression to determine whether any genes are significantly higher expressed in Bx Pos patients. AR (Bx Neg n = 38, Bx Pos n = 47), BIRC5 (Bx Neg n = 39, Bx Pos n = 45), ERG (Bx Neg n = 33, Bx Pos n = 41), PCA3 (Bx Neg n = 38, Bx Pos n = 46), TMPRSS2:ERG (Bx Neg n = 17, Bx Pos n = 31) and TMPRSS2 (Bx Neg n = 39, Bx Pos n = 45). All genes except AR were found to have significantly higher expression compared to Bx Neg patients. Solid bars—Bx Neg, Lined bars—Bx Pos. Data presented as RQ ± SEM. b) Correlation between the expression of TMPRSS2:ERG and ERG was determined using delta Ct against KLK3 in Bx Pos (black) and Bx Neg (grey) patients. c) Correlation between the expression of TMPRSS2:ERG and PCA3 was determined using delta C_t_ against KLK3 in Bx Pos (black) and Bx Neg (grey) patients. delta C_t_ = C_t gene of interest_ − C_t KLK3_.

**Fig 4:**
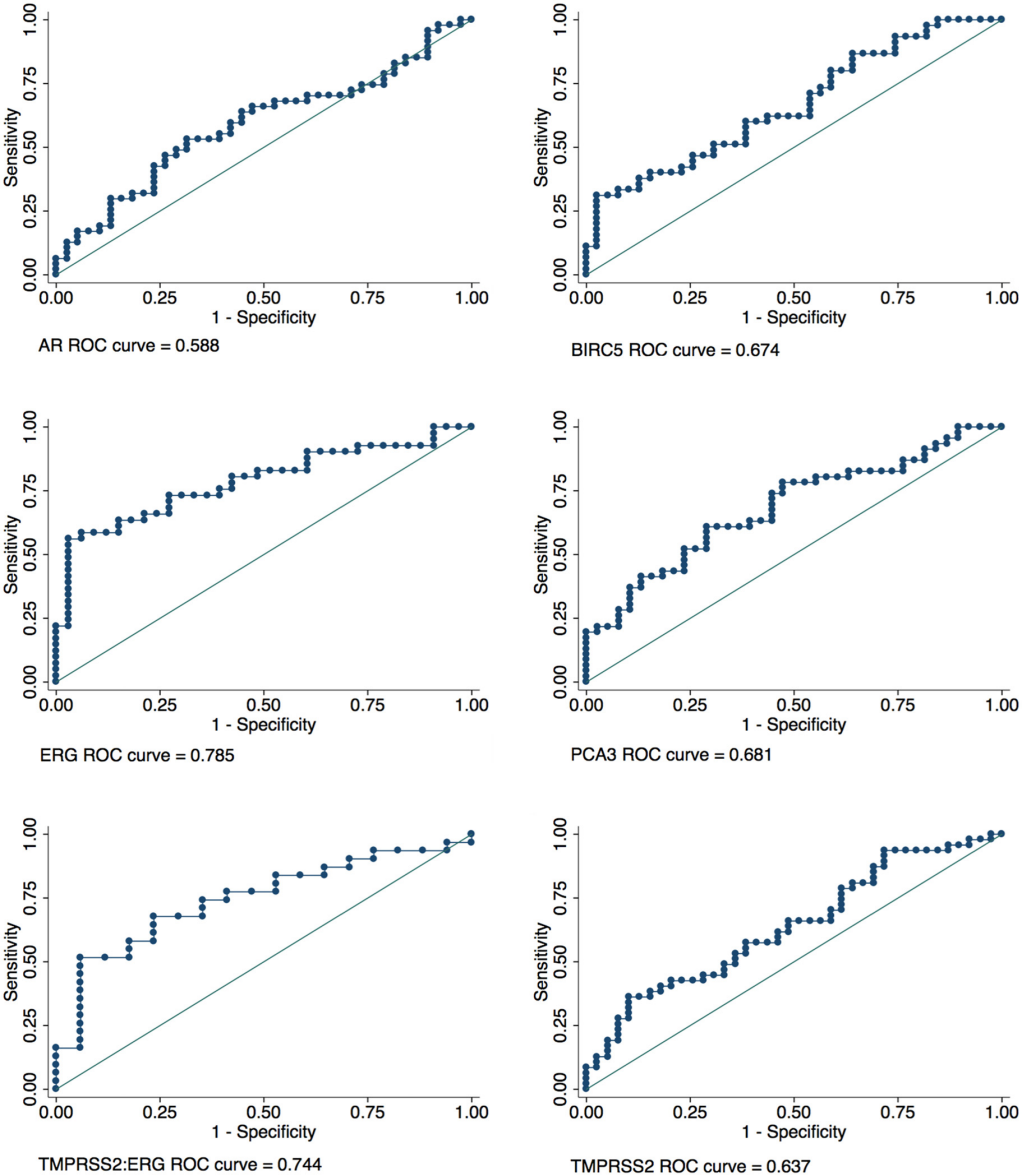
ROC Analysis of Prostate Related Genes. Individual ROC curves for AR, BIRC5, ERG, PCA3, TMPRSS2:ERG and TMPRSS2. T:E—TMPRSS2:ERG. AR (Bx Neg n = 38, Bx Pos n = 47), BIRC5 (Bx Neg n = 39, Bx Pos n = 45), ERG (Bx Neg n = 33, Bx Pos n = 41), PCA3 (Bx Neg n = 38, Bx Pos n = 46), TMPRSS2:ERG (Bx Neg n = 17, Bx Pos n = 31) and TMPRSS2 (Bx Neg n = 39, Bx Pos n = 45).

To determine whether urinary EVs could be used to examine other genes associated with prostate cancer we investigated the differential expression of androgen receptor (AR), BIRC5, ERG, PCA3, TMPRSS2:ERG and TMPRSS2 in Bx Pos versus Bx Neg patients (Fig 3a). Analysis of RQ showed that all genes except AR were significantly increased in Bx Pos patients versus Bx Neg patients (see S5 Fig for box plot distribution of delta C_t_). Analysis of the correlation between ERG and TMPRSS2:ERG expression (Fig 3b) revealed that there was a strong trend between the two genes (R^2^ = 0.826) consistent with urinary cell and tissue data suggesting that the most common fusion (TMPRSS2 (exon 1) with ERG (exon 4)) may give rise to increased ERG expression [12,18]. This trend was not evident when TMPRSS2:ERG expression was correlated with PCA3 expression (R^2^ = 0.110) (Fig 3c). ROC analyses were also carried out to determine the ability of these genes to discriminate biopsy status (Fig 4). Results confirmed that BIRC5, ERG, PCA3, TMPRSS2:ERG and TMPRSS2 were able to differentiate Bx Pos from Bx Neg patients (Table 4).

**Table 4:** ROC analysis of biopsy negative versus biopsy positive patients using urinary EV exoRNA.

				Asymptotic Normal	
Gene	*n*	ROC Area	Std. Err.	[95% Conf. Interval]	
AR	85	0.588	0.062	0.466	0.710
BIRC5	84	0.674	0.058	0.560	0.788
ERG	74	0.785	0.054	0.680	0.890
PCA3	84	0.681	0.058	0.567	0.795
TMPRSS2:ERG	48	0.744	0.073	0.600	0.888
TMPRSS2	86	0.637	0.060	0.519	0.754

Androgen receptor (AR), baculoviral IAP repeat containing 5 (BIRC5), Ets Related Gene (ERG), prostate cancer antigen 3 (PCA3), transmembrane protease serine 2 (TMPRSS2).

## Discussion

Analysis of RNA in urine has important implications for the development of non-invasive tests that examine differential gene expression and mutation detection. Despite the enormous potential that non-invasive analysis of underlying disease may have in guiding a physician, a key limitation to the use of biofluids is whether they accurately reflect gene expression changes in the parent tissue. Our analysis has utilized urinary EV exoRNA to determine whether it is: 1) reflective of tissue gene expression, and 2) able to differentiate BxPos from BxNeg patients non-invasively. Due to the ~25% inherent false negative rate of TRUS-guided prostate biopsy in detecting cancer and the limited amount of tissue surveyed during biopsy, we chose to utilize patients scheduled to undergo RP in order to compare the concordance between TMPRSS2:ERG mRNA detection in tissue versus in urine EVs. TMPRSS:ERG detection was specifically chosen to determine this correlation due to its known specificity to prostate tissue. Urinary exoRNA analysis was able to non-invasively detect tissue expression of TMPRSS2:ERG mRNA with very good accuracy and an overall concordance of 80%. In some cases, TMPRSS2:ERG expression was detected in ‘benign’ tissue regions. This may be due to the presence of micro foci of tumor in subsequent sections of the tissue extracted for RNA analysis below that originally analyzed by the pathologist (See S3 Fig). This is an inherent problem with this type of RP tissue analysis but may explain why some ‘benign’ regions unexpectedly showed positive TMPRSS2:ERG expression.

Three of the 21 patients had detectable TMPRSS2:ERG expression in tissue but undetectable TMPRSS2:ERG in the urinary EVs. Upon further analysis of this data it may be suggested that low-level expression in tissue may not be detected in EVs and may be viewed as a potential limitation. However, this should be considered with caution as there were only three such samples available for analysis and a larger study would need to be conducted to support this conclusion.

In one case, TMPRSS2:ERG was detected in the urinary EVs but was absent in the tissue available for analysis. Since whole-mount prostate sections were not available for total gland analysis, it is possible that the patient may have had a TMPRSS2:ERG positive focus in a tissue region unavailable for analysis. This highlights the potential advantage of urinary EVs as they may reflect a greater proportion of the prostate volume than what is obtainable via other sampling methods such as biopsy.

The ability to use random urine samples (collected without prostate massage) allowed us to analyze a wide range of subjects including young, healthy males (<35 years old) which indicated, as anticipated, that very few had detectable TMPRSS2:ERG levels (5% (2/40)). This was in contrast to older males in the Bx Pos (66%), Bx Neg (44%) and No Bx (41%) patient groups. This suggests that the likelihood of a TMPRSS2:ERG fusion event occurring may increase over time (i.e. with age), consistent with previous reports that prostate cancer incidence also increases with age [27]. Our detection rate of TMPRSS2:ERG via exoRNA in Bx Pos patients, was at the top of the range for that previously reported for TMPRSS2:ERG detection via urinary cell analysis in prostate cancer positive patients which ranged from 37–69% (22/32 (69%) [28], 10/15 (67%) [26], 8/19 (42%) [21], 56/138 (41%) [22], 29/78 (37%) [25], 9/13 (69%) [29]) with detection rates as low as 10/42 (24%) when no prostate massage was performed prior to urine collection [29] and 46/196 (24%) when a TMPRSS2:ERG detection cut-off of >10 copies was applied [30]. In patients without prostate cancer (determine via negative biopsy), the detection of TMPRSS2:ERG via urinary cell analysis was lower than our detection rate via EV analysis and ranged from 7–21% (2/30 (7%) [25], 4/30 (13%) [26], 4/24 (17%) [28], 20/96 (21%) [22] and as low as 6% without prior prostate massage, 15/247 (6%) [29]. While there was a large number of Bx Neg and No Bx patients with a detectable TMPRSS2:ERG fusion event in our current study, TMPRSS2:ERG expression level (analyzed via RQ) was highest in the Bx Pos group consistent with the confirmed presence of cancer. Low-level expression in the Bx Neg and No Bx groups may represent spurious RNA transcripts rather than contamination as there was a complete absence of TMPRSS2:ERG exoRNA (0% (0/40)) in the 40 post-radical prostatectomy patients tested. The use of an expression cut-off such as that employed by Leyten et al, [30] could potentially allow for the differentiation between low-level spurious gene expression (such as that seen in Bx Neg and age-matched No Bx patients) and stable gene expression observed in Bx Pos patients. One can only speculate as to why EV analysis detects more positive TMPRSS2:ERG expressing patients than the analysis of urinary cells particularly in the No Bx and Bx Neg groups. One suggestion may be because EVs represent a greater survey of the prostate as they are constitutively released by potentially all cells. This is in contrast to the analysis of prostate derived urinary cells which are turned over at a much slower rate. Thus the analysis of EVs in Bx Neg (and No Bx) groups may give a greater opportunity to detect spurious TMPRSS2:ERG reads as compared to urinary prostate cell analysis.

ERG gene expression levels were also examined to determine whether a correlation between exoRNA expression of TMPRSS2:ERG and ERG was evident. Urinary EV results strongly supported previous reports that increased ERG expression may be associated with the TMPRSS2:ERG fusion event [12,18,31]. Analysis of ERG expression was found to perform better than the analysis of TMPRSS2:ERG expression in differentiating Bx Pos from Bx Neg patients. This may be because TMPRSS2:ERG detected by our assay may account for only a fraction of ERG related fusion events [18]. Thus, our analysis of ERG expression alone may potentially pick up additional ERG fusion events beyond detected by our TMPRSS2:ERG assay making ERG alone a more sensitive marker in our study.

To examine the use of EVs as a platform to non-invasively differentiate Bx Pos and Bx Neg patients without prostate massage, we analyzed the ability of other genes previously implicated in prostate cancer to differentiate Bx Pos and Bx Neg patients using the urinary EV platform. Relative quantitation analysis demonstrated that exoRNA for BIRC5, ERG, PCA3 and TMPRSS2:ERG standardized to KLK3, differentiated Bx Pos from Bx Neg patients. AR expression was unable to differentiate these groups. This trend was mirrored in the ROC analysis of these genes. It is difficult to postulate why documented changes in AR expression in cancer was not mirrored in our exoRNA study except that perhaps AR expression is not governed at the mRNA level but rather at the protein level.

This pilot study demonstrates the potential utility of exoRNA to examine prostate-related gene expression isolated from a single, random urine sample. Specifically, EV analysis has an ~80% accuracy for the detection of TMPRSS2:ERG mutation, assuming no false negatives in the radical prostatectomy samples, and is influenced by level of gene expression in tissue. The ability to segregate Bx Pos from Bx Neg patients using a non-invasive urine sample without the use of a prostate massage was also demonstrated, and supports the future development of a potential EV based urinary diagnostic test for prostate cancer. Larger cohorts are now needed to confirm these studies. Finally, due to the ubiquitous release of EVs from cells, it is possible that urinary EVs may become a novel, non-invasive platform to examine other organs of the genitourinary tract, including the kidney and bladder.

## Supporting Information

S1 Fig Cartoon of Prostate Tissue Sections Taken for Comparative Analysis of TMPRSS2:ERG Gene Expression in Tissue Versus Urinary exoRNA. Cartoons showing benign (blue) and cancer (pink) regions from a radical prostatectomy that were selected for RNA extraction and gene expression analysis. Tumor and benign regions positive for TMPRSS2:ERG expression are indicated with a Red ‘+’. Negative TMPRSS2:ERG expression is indicated with a Blue ‘-’. Purple ‘+’ is used to indicate positive TMPRSS2:ERG expression in a pooled sample of tissue. T:E—TMPRSS2:ERG, A—anterior, P—posterior, L—left, R—right. ‘Level’ indicates the area of the prostate surveyed for gene expression analysis (urethra to seminal vesicles).
(TIFF)

S2 Fig Detection of mRNA Transcripts in FFPE Tissue. Agilent bioanalyzer DNA chip analysis of amplicons generated via PCR in no template control (NTC) and samples with reverse transcriptase reaction (RT) and without reverse transcriptase (No RT) reaction. The ‘No RT’ samples failed to generate amplicons consistent with the RNA specific primers and probe for each gene. Samples undergoing RT reaction generated amplicons of similar size to the ABI reported amplicon size. NTC failed to generate amplicons suggesting no contamination. ABI predicted amplicon size: TMPRSS2:ERG 106 bp, KLK3 64 bp, PCA3 80 bp, BIRC5 93 bp, ERG 104 bp. Bioanalyzer estimated amplicon size: TMPRSS2:ERG 116 bp, KLK3 70 bp, PCA3 81 bp, BIRC5 89 bp, ERG 115 bp. KLK3—prostate specific antigen, PCA3—prostate cancer antigen 3, BIRC5—baculoviral IAP repeat containing 5, ERG—Ets Related Gene.
(TIFF)

S3 Fig Representative H&E Stained Tissue of the Various Benign and Tumor Areas Surveyed for TMPRSS2:ERG Expression. Prostatectomy tissue (5um) was stained with H&E to demonstrate the morphology of each area. Analysis of sections serial to those originally analyzed by the pathologist revealed in some cases micro foci of tumor (see section C12), which may explain TMPRSS2:ERG expression in previously indicated benign regions.
(TIFF)

S4 Fig Box plot Analysis of TMPRSS2:ERG delta C_t_ Expression in Urinary EVs. Box plot analysis demonstrating the spread of TMPRSS2:ERG delta Ct in Bx Neg, Bx Pos, Cont, No Bx and RP groups. Delta Ct was determined as C_t TMPRSS2:ERG_ − C_t KLK3_. Bx Neg—biopsy negative (n = 39), Bx Pos—biopsy positive (n = 47), Cont—control males <35 years old (n = 40), No Bx—no biopsy (age matched control) (n = 44), RP—radical prostatectomy (n = 37).
(TIFF)

S5 Fig Box plot Analysis of Prostate Cancer Related Gene delta C_t_ Expression in Urinary EVs. Box plot analysis demonstrating the Delta C_t_ spread of prostate cancer related genes AR, BIRC5, ERG, PCA3, T:E, and TMPRSS in Bx Pos and Bx Neg patients. Delta C_t_ was determined as C_t gene of interest_ − C_t KLK3_. AR (Bx Neg n = 38, Bx Pos n = 47), BIRC5 (Bx Neg n = 39, Bx Pos n = 45), ERG (Bx Neg n = 33, Bx Pos n = 41), PCA3 (Bx Neg n = 38, Bx Pos n = 46), TMPRSS2:ERG (Bx Neg n = 17, Bx Pos n = 31) and TMPRSS2 (Bx Neg n = 39, Bx Pos n = 45).
(TIFF)
