# Supplementary figures and images for: Urine Exosomes for Non-Invasive Assessment of Gene Expression and Mutations of Prostate Cancer
Source: PLoS One. 2016 May 4;11(5):e0154507. doi: 10.1371/journal.pone.0154507 (PMC4856378; doi:10.1371/journal.pone.0154507)

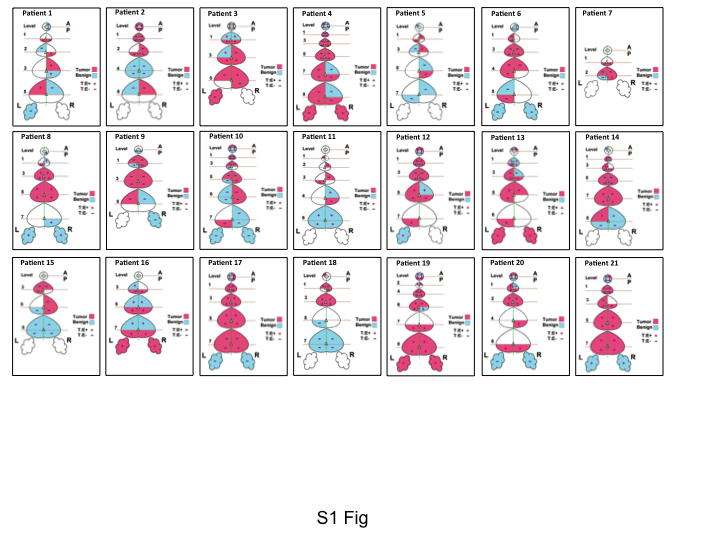

Supplement: S1 Fig — Cartoons showing benign (blue) and cancer (pink) regions from a radical prostatectomy that were selected for RNA extraction and gene expression analysis. Tumor and benign regions positive for TMPRSS2:ERG expression are indicated with a Red ‘+’. Negative TMPRSS2:ERG expression is indicated with a Blue ‘-’. Purple ‘+’ is used to indicate positive TMPRSS2:ERG expression in a pooled sample of tissue. T:E—TMPRSS2:ERG, A—anterior, P—posterior, L—left, R—right. ‘Level’ indicates the area of the prostate surveyed for gene expression analysis (urethra to seminal vesicles). (TIFF) [file pone.0154507.s001.tiff]

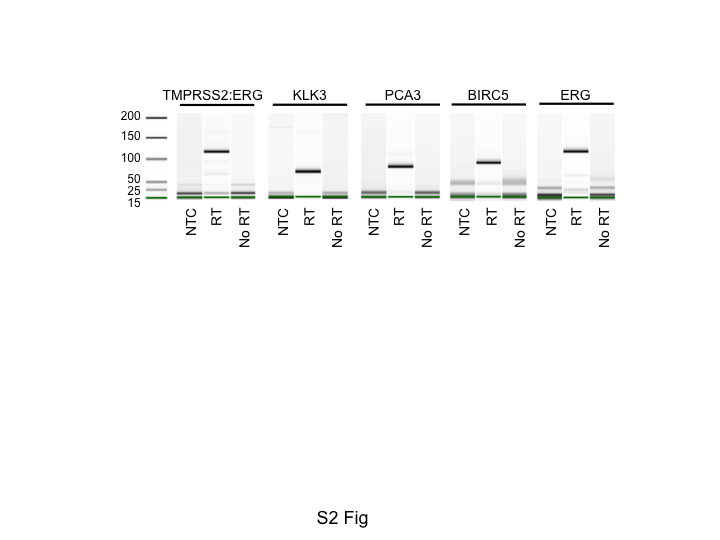

Supplement: S2 Fig — Agilent bioanalyzer DNA chip analysis of amplicons generated via PCR in no template control (NTC) and samples with reverse transcriptase reaction (RT) and without reverse transcriptase (No RT) reaction. The ‘No RT’ samples failed to generate amplicons consistent with the RNA specific primers and probe for each gene. Samples undergoing RT reaction generated amplicons of similar size to the ABI reported amplicon size. NTC failed to generate amplicons suggesting no contamination. ABI predicted amplicon size: TMPRSS2:ERG 106 bp, KLK3 64 bp, PCA3 80 bp, BIRC5 93 bp, ERG 104 bp. Bioanalyzer estimated amplicon size: TMPRSS2:ERG 116 bp, KLK3 70 bp, PCA3 81 bp, BIRC5 89 bp, ERG 115 bp. KLK3—prostate specific antigen, PCA3—prostate cancer antigen 3, BIRC5—baculoviral IAP repeat containing 5, ERG—Ets Related Gene. (TIFF) [file pone.0154507.s002.tiff]

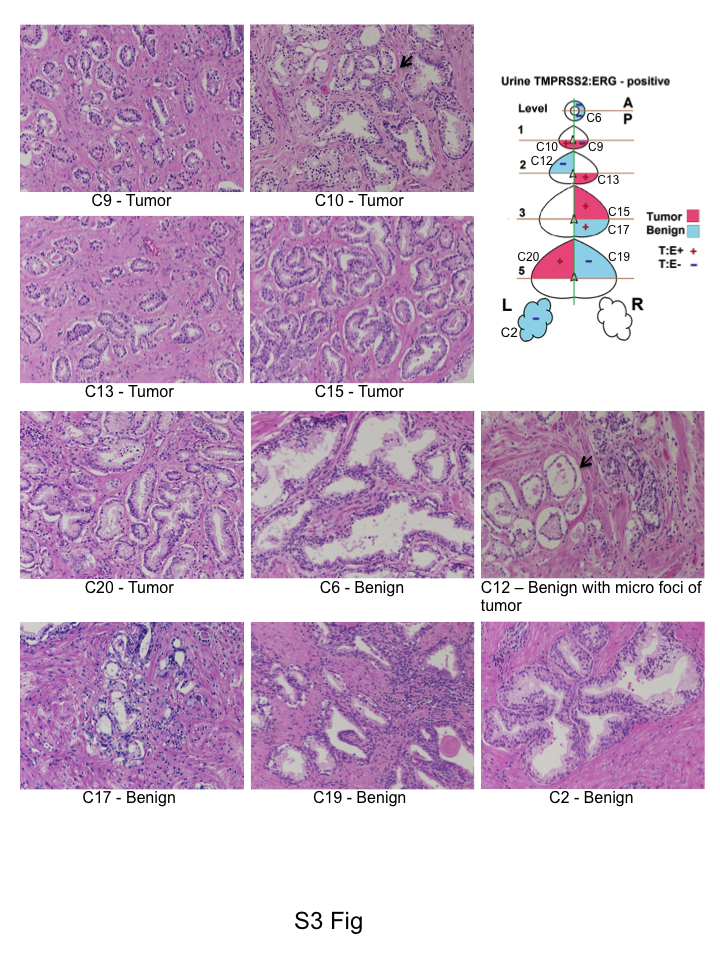

Supplement: S3 Fig — Prostatectomy tissue (5um) was stained with H&E to demonstrate the morphology of each area. Analysis of sections serial to those originally analyzed by the pathologist revealed in some cases micro foci of tumor (see section C12), which may explain TMPRSS2:ERG expression in previously indicated benign regions. (TIFF) [file pone.0154507.s003.tiff]

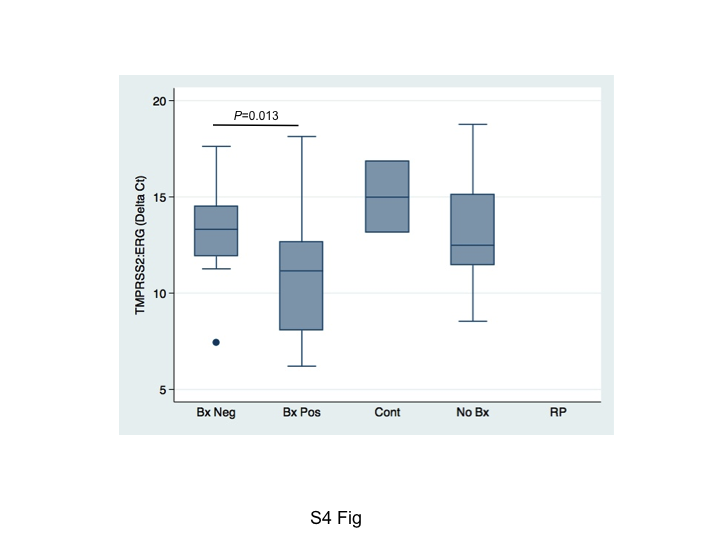

Supplement: S4 Fig — Box plot analysis demonstrating the spread of TMPRSS2:ERG delta Ct in Bx Neg, Bx Pos, Cont, No Bx and RP groups. Delta Ct was determined as Ct TMPRSS2:ERG − Ct KLK3. Bx Neg—biopsy negative (n = 39), Bx Pos—biopsy positive (n = 47), Cont—control males <35 years old (n = 40), No Bx—no biopsy (age matched control) (n = 44), RP—radical prostatectomy (n = 37). (TIFF) [file pone.0154507.s004.tiff]

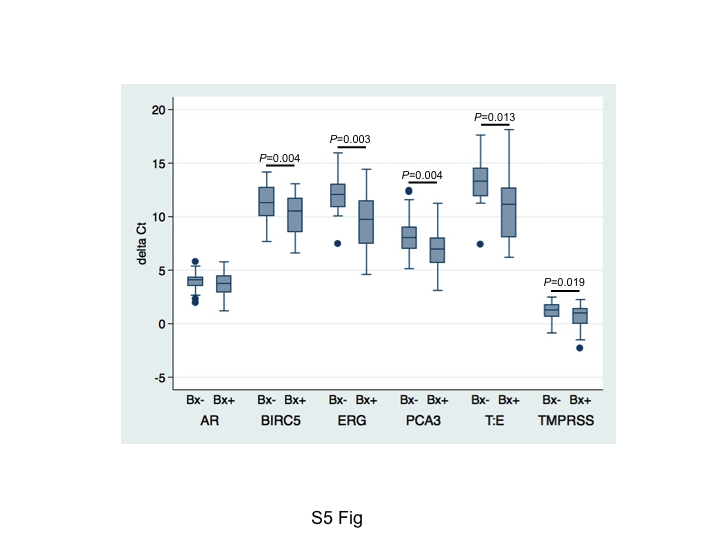

Supplement: S5 Fig — Box plot analysis demonstrating the Delta Ct spread of prostate cancer related genes AR, BIRC5, ERG, PCA3, T:E, and TMPRSS in Bx Pos and Bx Neg patients. Delta Ct was determined as Ct gene of interest − Ct KLK3. AR (Bx Neg n = 38, Bx Pos n = 47), BIRC5 (Bx Neg n = 39, Bx Pos n = 45), ERG (Bx Neg n = 33, Bx Pos n = 41), PCA3 (Bx Neg n = 38, Bx Pos n = 46), TMPRSS2:ERG (Bx Neg n = 17, Bx Pos n = 31) and TMPRSS2 (Bx Neg n = 39, Bx Pos n = 45). (TIFF) [file pone.0154507.s005.tiff]
